# LightMixer: A novel lightweight convolutional neural network for tomato disease detection

**DOI:** 10.3389/fpls.2023.1166296

**Published:** 2023-05-09

**Authors:** Yi Zhong, Zihan Teng, Mengjun Tong

**Affiliations:** ^1^ College of Mathematics and Computer Science, Zhejiang A&F University, Hangzhou, China; ^2^ School of Design, The Hong Kong Polytechnic University, Hong Kong, Hong Kong, SAR, China

**Keywords:** tomato leaf disease, lightweight model, convolutional neural networks, deep learning, disease detection

## Abstract

Tomatoes are among the very important crops grown worldwide. However, tomato diseases can harm the health of tomato plants during growth and reduce tomato yields over large areas. The development of computer vision technology offers the prospect of solving this problem. However, traditional deep learning algorithms require a high computational cost and several parameters. Therefore, a lightweight tomato leaf disease identification model called LightMixer was designed in this study. The LightMixer model comprises a depth convolution with a Phish module and a light residual module. Depth convolution with the Phish module represents a lightweight convolution module designed to splice nonlinear activation functions with depth convolution as the backbone; it also focuses on lightweight convolutional feature extraction to facilitate deep feature fusion. The light residual module was built based on lightweight residual blocks to accelerate the computational efficiency of the entire network architecture and reduce the information loss of disease features. Experimental results show that the proposed LightMixer model achieved 99.3% accuracy on public datasets while requiring only 1.5 M parameters, an improvement over other classical convolutional neural network and lightweight models, and can be used for automatic tomato leaf disease identification on mobile devices.

## 1 Introduction

Tomatoes are one of the most widely grown and consumed crops worldwide and are a high source of income for many agricultural countries (Al‐Gaashani et al., 2022). According to the United Nations Food and Agriculture Organization, the global production of tomatoes alone is expected to exceed 180 million tons by 2020 (Nations, 2020). China is currently ranked first among tomato-producing countries (Faostat, 2022). However, tomatoes are susceptible to various diseases during cultivation, which can significantly affect their yield (Xu et al., 2022). The early identification and detection of diseases can reduce the infection and spread of tomato crops and reduce the use of unnecessary agrochemicals. Early disease characteristics are expressed on plant leaves, and farmers or pathologists with extensive experience in tomato cultivation can effectively diagnose the type of crop disease and take necessary measures through visual inspection of various infected leaves (Cap et al., 2021). Although such traditional methods of disease inspection have a positive effect on the prevention of tomato diseases, they are only usable by professionals or empiricists, especially in developing countries where farmers in many regions are often unable to make effective diagnoses of plant diseases. In addition, subjective factors in personal perception, inefficiency, and labor costs are also issues that should be considered (Abbas et al., 2021). Therefore, the design of a simple, efficient, and accurate automatic tomato leaf disease diagnosis system that requires few computational parameters is of great importance to help farmers detect tomato diseases and improve tomato production. A lightweight model called LightMixer is proposed in this study. This model uses lightweight convolution to extract disease features, passes them to the deep network, and introduces the designed residual blocks to further reduce the number of parameters of the network and accelerate the computational efficiency of the model. The contributions of this study are as follows:

1. A novel lightweight deep learning model called LightMixer was proposed for the automatic detection of tomato leaf diseases under field conditions.
2. The depth convolution with Phish (DCWP) and light residual (LR) modules were carefully designed to improve deep feature fusion while reducing the number of model parameters.
3. Phish activation function was used to replace the commonly used rectified linear unit function to reduce the information loss of tomato leaf disease features and improve the nonlinear expression of the model.
4. Compared with other classical convolutional neural network (CNN) models, the proposed model achieved minimum memory cost and highest accuracy, making it more suitable for deployment in mobile devices or small embedded devices.

## 2 Related work

In recent years, with the development of machine learning and deep learning, the extensive use of computer vision techniques in agriculture has achieved remarkable results, particularly in the field of plant disease identification. Bhatia et al. (2020) proposed a hybrid support vector machine and logistic regression algorithm to extract the disease features of tomato powdery mildew to diagnose tomato diseases, achieving a 92.37% identification accuracy. Bhagat et al. (2020) used a grid search technique to optimize the support vector machine algorithm to classify plant leaf diseases. Chopda et al. (2018) built an Android application based on a decision-tree algorithm to effectively solve the classification problem of cotton crop diseases for local farmers. However, traditional machine learning image processing algorithms require the manual construction of disease features, which increases labor costs and cannot be adapted to identify multiple types of plant diseases. Therefore, deep learning algorithms that do not require manual feature construction are increasingly used to identify and diagnose plant diseases. Darwish et al. (2020) achieved a 98.2% recognition accuracy on a dataset containing five types of maize leaf images based on a combination of an integrated VGGNet architecture and an orthogonal particle swarm optimization algorithm to overcome the limitations in sample size and diversity. Fan et al. (2022) investigated migration learning and feature fusion to recognize plant leaves; the experimental results showed that the accuracy of the method exceeded 97% on a publicly available dataset. Chen et al. (2020) proposed the INC-VGGN network architecture for rice disease recognition by fusing the VGGNet architecture with the Inception module and achieved good recognition accuracy. Karthik et al. (2020) designed an attention-based deep residual network to identify tomato diseases; experimental results showed that the designed network architecture was able to identify the disease types with 98% accuracy. Zhao et al. (2021) improved the ResNet network architecture based on the attention module to extract various disease features of tomato leaves, achieving an average recognition accuracy of 96.81%. Lee et al. (2020) explored the identification of plant diseases using a recurrent neural network incorporating an attention mechanism, which has better generalization over public datasets compared to the classical CNN approach. L Lakshmanarao et al. (2021) applied the Convnets network architecture to identify nine tomato diseases. Agarwal et al. (2020) pre-trained the VGG16 network architecture, compressed the selected model, and validated it on a public dataset; experimental results showed that their proposed network architecture achieved an accuracy of 98%. Liu et al. (2022) chose the ResNext50 network architecture as the backbone and introduced expanded convolution and CA attention modules to design a DCCAM-MRNet network to identify six tomato leaf diseases. The experimental results showed that the classification accuracy of DCCAM-MRNet reached 94.3% with fewer parameters than the ResNext50 network architecture, which has a notable performance advantage.

Although the aforementioned studies strongly demonstrate the effectiveness of CNN architectures in the field of plant disease identification, these architectures are inevitably problematic because of their large number of parameters and high computational complexity. Therefore, designs focusing on lightweight CNNs have been widely proposed. Kamal et al. (2019) constructed lightweight CNNs for plant disease recognition on public datasets based on deep separable convolution using the MobileNet architecture, which has fewer parameters compared to other classical CNNs. Chen et al. (2022) replaced the standard convolutional approach in the DenseNet architecture, and Singh et al. (2021) verified the effectiveness of their lightweight model by fine-tuning several pre-trained models and comparing them with other classical CNNs. Barman et al. (2020) proposed the use of the MobileNet and SSCNN architectures to detect citrus leaf diseases in stages to achieve the efficiency of lightweight models. The experimental results showed that the algorithm had lower computational complexity and achieved a high accuracy of 99%. With similar accuracy, Bi et al. (2022) demonstrated a lower computational complexity using a MobileNet network architecture with deeply separable convolutions to identify two classes of apple diseases. The use of superior performance loss functions to optimize network architectures has also been proposed for the development of lightweight deep learning network architectures. For example, Zeng et al. (2022) used a new loss function to optimize the proposed lightweight LDSNet network architecture to recognize corn disease images based on a public dataset. The experimental results showed that the optimized network architecture achieved an accuracy of 95.4%, outperforming other classical lightweight models. Chen et al. (2021) used an enhanced loss function to optimize the MobileNetV2 model to identify rice diseases in complex backgrounds; they achieved an average accuracy of 98.48%, outperforming other classical methods. The aforementioned studies performed well in the field of plant disease identification, but the lightness of the model and accuracy of identification can be further explored.

## 3 Materials and methods

### 3.1 Image dataset acquisition and pre-processing

The data in this study were obtained from the PlantVillage public dataset (Arun Pandian and Gopal, 2019), which has 18835 tomato leaf images classified into ten categories, one healthy category and nine categories corresponding to different tomato leaf diseases. **Table 1** shows the details of the dataset. **Supplementary Figure 1** shows sample images for each tomato leaf category and describes the relevant information in the dataset, including the category labels, names of the diseases, and number of images used in the study for each category. To validate the generalization of the deep learning model, the image dataset in this study was randomly divided into an 80% training set, 10% validation set, and 10% test set.

**Table 1:** Details of the dataset.

Category	Disease Name	Image (Number)
1	Bacterial spot	2127
2	Early blight	1000
3	Healthy	1591
4	Late blight	1909
5	Leaf mold	1000
6	Septoria leaf spot	1771
7	Two-spotted spider mite	1676
8	Target spot	1404
9	Mosaic virus	1000
10	Yellow Leaf Curl Virus	5357

For a deep learning model, the more training data input into the model, the better the model learns. Therefore, several data enhancement methods were used to perform data enhancement on the training dataset before the deep learning model started training, including random image cropping, horizontal random flip, vertical random flip, and random rotation. All images were resized to 224 × 224 pixels. **Supplementary Figure 2** shows the results obtained after applying the data enhancement methods to the tomato leaf images in the training set.

### 3.2 LightMixer model

**Figure 1:**
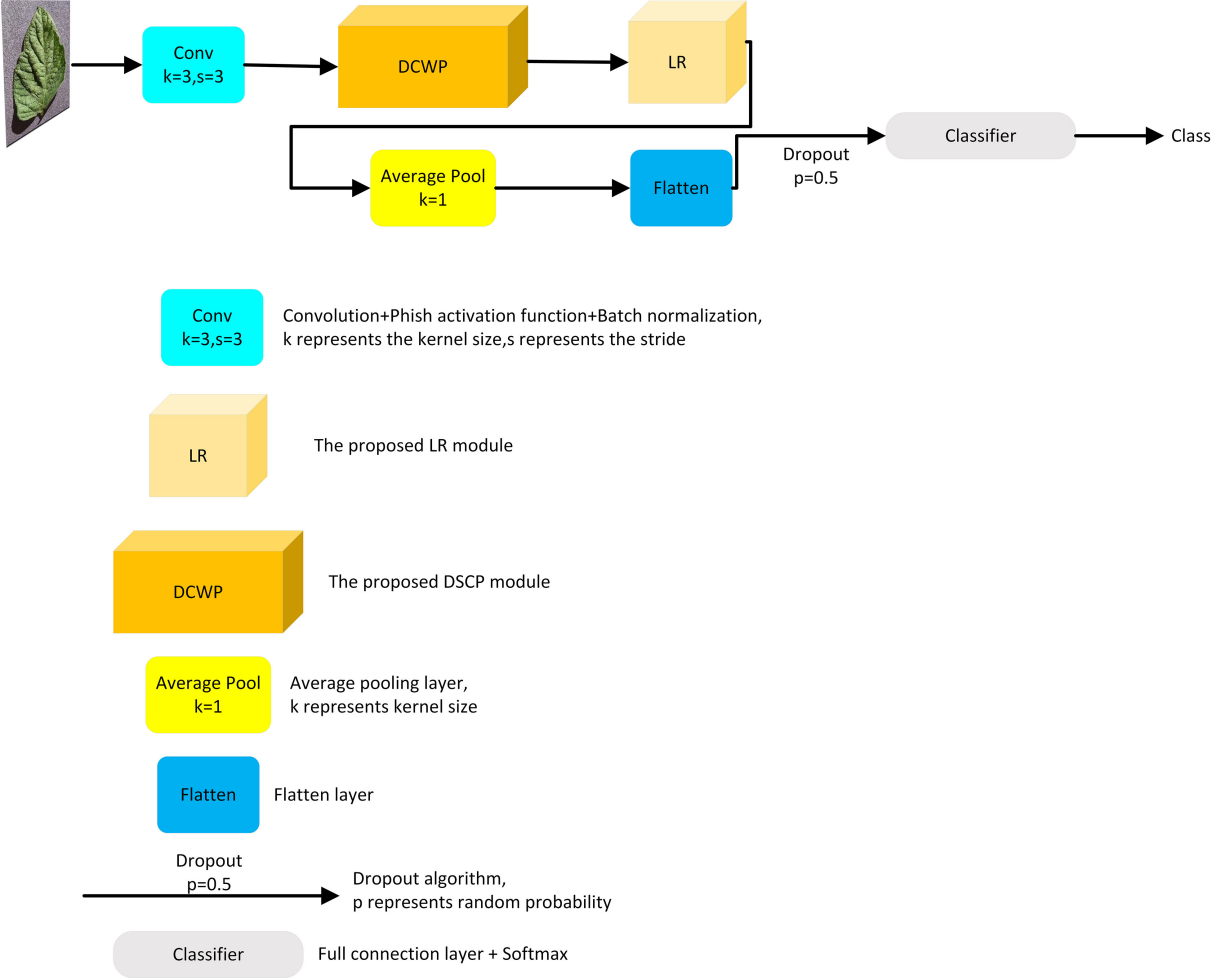
The structure of the LightMixer model.

In this study, a lightweight CNN model called LightMixer is proposed for identifying tomato leaf diseases in complex environments. The proposed LightMixer model focuses on both lower memory cost and higher accuracy to address the migration difficulties for mobile or small embedded devices. By designing and assembling the DCWP and LR modules, LightMixer can accurately extract features from tomato leaf disease images with complex backgrounds and achieve high accuracy with low-memory cost. LightMixer is a lightweight CNN model that is suitable for mobile and cross-connected wireless communication devices. The LightMixer structure is shown in **Figure 1**. It mainly consists of a convolutional block, a DCWP module, an LR module, a mean pooling layer, a Flatten layer, and a fully connected layer. A 3 × 3 convolutional layer is used in the first convolutional block of LightMixer to obtain a rich feature representation of the image. After the first convolution block, the DCWP module is connected to extract the deep feature information of the feature map without parameter redundancy. The LR module is connected after the DCWP module to increase the depth of the CNN with a small number of parameters to improve the nonlinear representation of the model while reducing the number of network parameters. The average pooling layer is used to reduce the spatial dimensionality of the feature map and increase the perceptual field of the model. The last classifier block uses the softmax function to implement disease classification. The configuration of the LightMixer model is listed in **Table 2**.

**Table 2:** Architecture of the LightMixer model.

Layer	Type	Output Shape	Parameters
Input	Input	[3,224,224]	0
	Conv2d	[32, 3, 75, 75]	30
DCWP Module	Conv2d	[32, 224, 75, 75]	896
	DepthWiseConv	[32, 224, 75, 75]	0
	Phish	[32, 224, 75, 75]	0
	Conv2d	[32, 224, 74, 74]	200,928
	BatchNorm2d	[32, 224, 74, 74]	448
LR Module	Conv2d	[32, 224, 74, 74]	18,368
	Phish	[32, 224, 74, 74]	0
	BatchNorm2d	[32, 224, 74, 74]	448
	Conv2d	[32, 224, 74, 74]	50400
	Phish	[32, 224, 74, 74]	0
	BatchNorm2d	[32, 224, 74, 74]	448
Average pool	AdaptiveAvgPool2d	[32, 224, 1, 1]	0
Flatten	Flatten	[32, 224]	0
Dropout	Dropout	[32, 224]	0
Linear	Linear	[32, 10]	2250

### 3.3 Phish activation function

The activation function is a key component for minimizing the loss in deep neural networks and can both enhance the nonlinear representation and accelerate the computational efficiency of the entire model, thus improving the accuracy of tomato leaf disease identification. Compared with traditional activation functions introduced in CNN models, the Phish activation function outperforms most traditional activation functions in terms of classification; therefore, it is introduced in this study (Naveen, 2022). The Phish activation function consists of the existing GELU function (Hendrycks and Gimpel, 2016) with the Tanh function, and is defined as follows:


(1) Phish(x)=xTanh(GELU(x))

where x denotes the input, and the mathematical operations of the Tanh and GELU functions are shown in Equations (2) and (3), respectively.


(2) $$Tanh\left(x\right)=\frac{e^{x}-e^{-x}}{e^{-x}+e^{x}}$$

(3) $$GELU\left(x\right)=\frac{\pi }{2}x\left(1+tanh^{-1}\left[\sqrt{\frac{2}{\pi }}\left(x+0.044715x^{3}\right)\right]\right)$$

Therefore, by combining Equations (1), (2), and (3), the equation for the Phish function can be expressed as:


(4) $$Phish\left(x\right)=x\frac{e^{\frac{\pi }{2}x\left(1+tanh^{-1}\left[\sqrt{\frac{2}{\pi }}\left(x+0.044715x^{3}\right)\right]\right)}-e^{-\;\frac{\pi }{2}x\left(1+tanh^{-1}\left[\sqrt{\frac{2}{\pi }}\left(x+0.044715x^{3}\right)\right]\right)}}{e^{-\;\frac{\pi }{2}x\left(1+tanh^{-1}\left[\sqrt{\frac{2}{\pi }}\left(x+0.044715x^{3}\right)\right]\right)}+e^{\frac{\pi }{2}x\left(1+tanh^{-1}\left[\sqrt{\frac{2}{\pi }}\left(x+0.044715x^{3}\right)\right]\right)}}$$

where x denotes the input and Phish(x) denotes the nonlinear output after mathematical operations.

### 3.4 Depth convolution with phish

**Figure 2:**
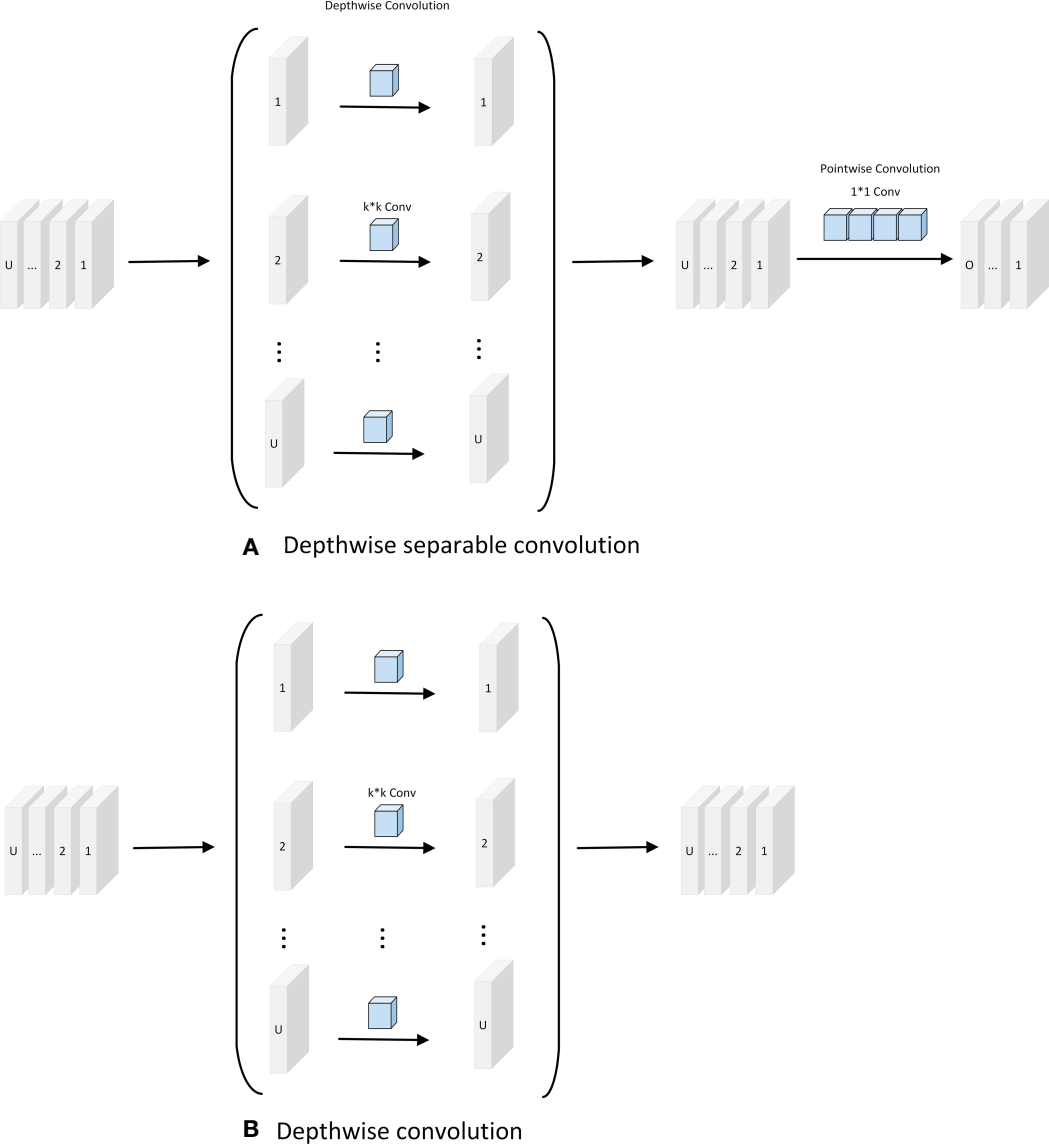
Depth separable convolution and Depthwise convolution. **(A)** Depthwise separable convolution; **(B)** Depthwise convolution.

Depth-separable convolution reduces the number of parameters required for convolution computation by splitting the correlation between the spatial and channel dimensions; it has also been shown in some studies to improve the efficiency of convolution kernel parameters. As shown in **Figure 2A**, the standard convolution of the k × k kernel increases the number of channels in the feature map. Depthwise convolution expands the perceptual field of the network without changing the number of channels and extracts deeper features of the feature map. The output feature map of the point-by-point convolution is added point-by-point to the input feature map to obtain the output feature map. As shown in **Figure 2B**, depthwise convolution is formed based on depth-separable convolution, which has a lower number of parameters and lower cost compared to depth-separable convolution. As shown in **Figure 2**, assuming that U and O are the numbers of input and output channels, respectively, and $k^{2}$ is the size of the convolution kernel, the number of parameters of depthwise separable convolution is

$k^{2}\times U+U\times O$ , and the number of parameters of depthwise convolution is

$k^{2}\times U$ . Therefore, the ratio of parameters can be calculated as follows:


(5) $$\frac{k^{2}\times U+U\times O}{k^{2}\times U}=1+\frac{O}{k^{2}}$$

**Figure 3:**
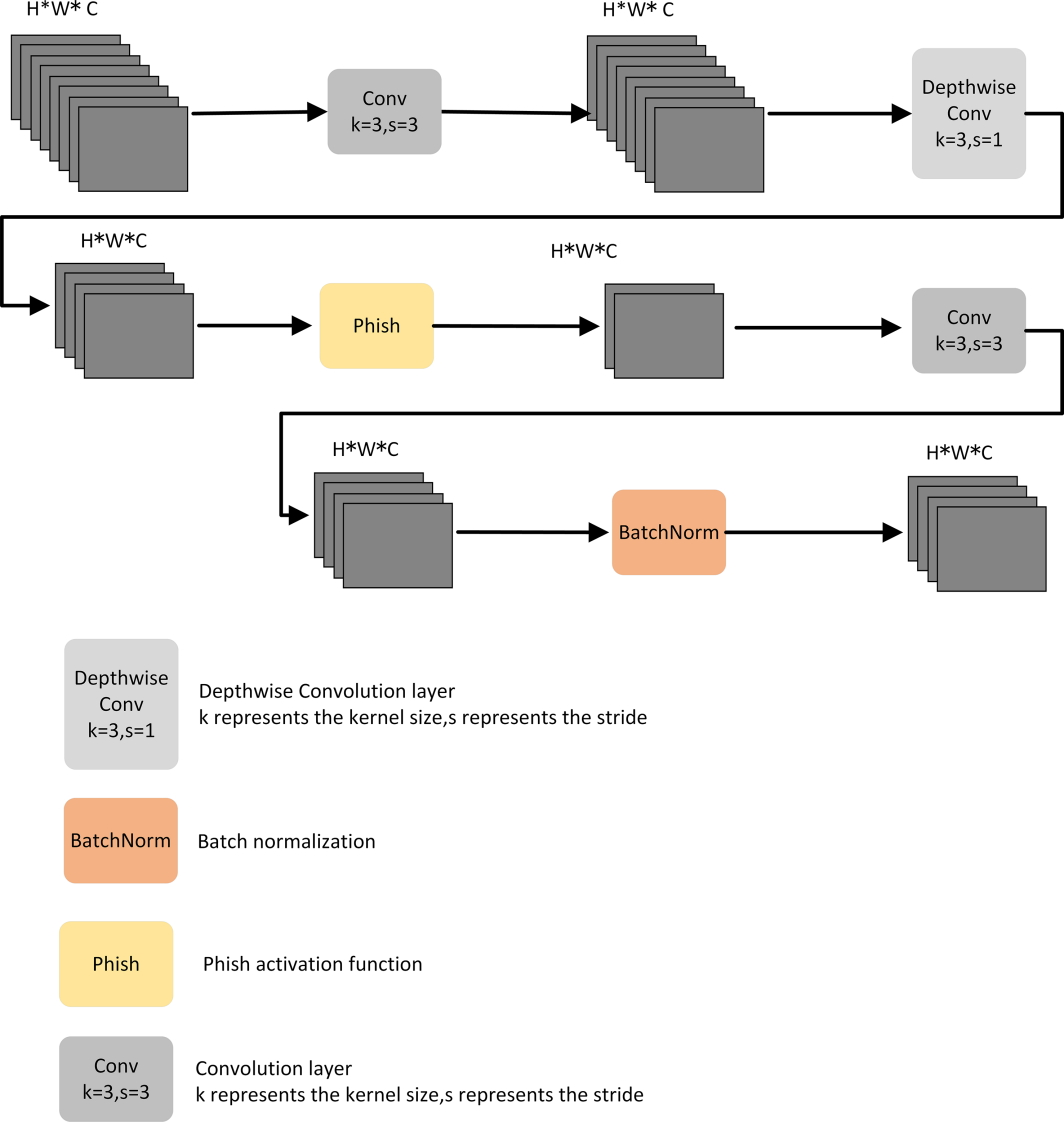
Depth Convolution with Phish Module.

In this study, the DCWP module was designed with depthwise convolution as the backbone. To avoid missing feature information in the feature map compression process, no nonlinear activation function was used, and the Phish function was used to splice after the depthwise convolution to make full use of the relevant information in the feature map to transfer the shallow feature information to the deep layer. The DCWP module has been designed to be used as a tool for deeper layers; its structure is shown in **Figure 3**.

### 3.5 Light residual module

To address the challenge of difficult deployment of CNN models for tomato leaf disease identification in mobile terminals and low-memory devices, there is an urgent need to design a new network architecture that guarantees high accuracy in identifying tomato leaf diseases in complex contexts while further reducing the total number of parameters and model size. We designed a residual-based LR module to make the model more lightweight and improve its deployment capability.

**Figure 4:**
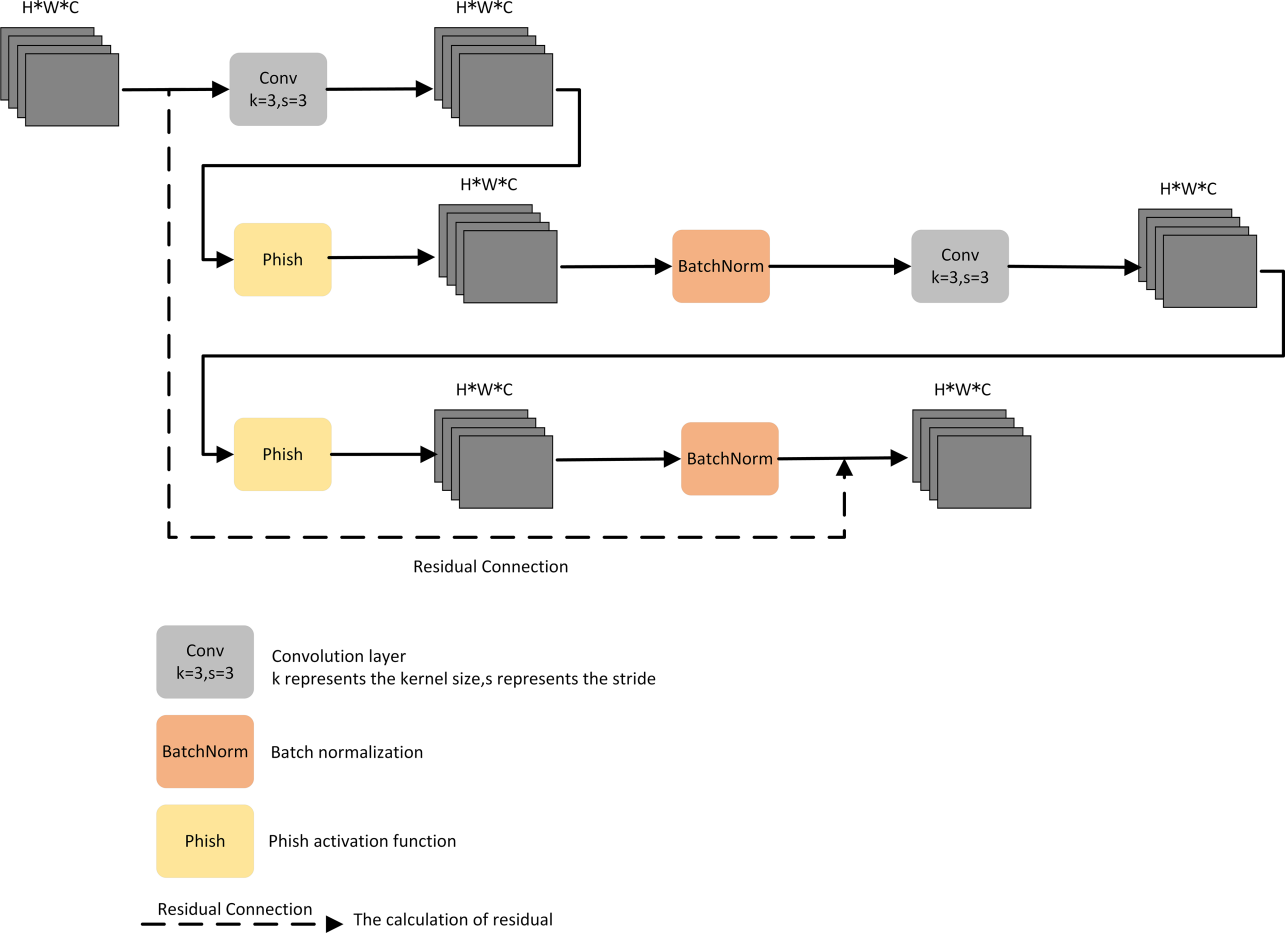
Light Residual Module.

First, a convolutional layer and the Phish activation function were connected, and a batch normalization layer was added after the Phish activation function, as the network depth poses a challenge to the convergence speed of the entire network. Then, the sequentially connected convolutional layers, Phish activation function, and batch normalization layer were multiplexed in series to effectively improve the feature extraction for tomato leaf disease. Finally, residual connections constructed efficient residual blocks, which improved the ability of gradient propagation across layers and could prevent gradients in deep convolutional layers from vanishing. The structure of the LR module is shown in **Figure 4**.

## 4 Experimental results and discussion

### 4.1 Hyperparameters and experimental equipment

The hyperparameter settings used in the experiments are presented in **Table 3**. The programming language used for the experiments was Python, and PyTorch was used as the deep learning framework. A detailed configuration of the development environment is presented in **Table 4**.

**Table 3:** Hyperparameter configuration of CNN.

Hyperparameter	Value
Loss function	Cross-entropy
Epochs	70
Learning rate	0.0001
Optimizer	Adam

**Table 4:** Software and hardware configuration.

Hardware & Software Device	Model
CPU	Intel I5-10400H
GPU	Nvidia 3060Ti
RAM	16GB
CUDA	11.0
Operating system	Windows 10

To evaluate the performance of the model, recall, accuracy, precision and F1-score were selected as the metrics for comprehensive evaluation. The mathematical expressions for the evaluation metrics are as follows:


(5) $$Recall=\frac{TP}{TP+FN}$$

(6) $$Accuracy=\frac{TP+TN}{TP+TN+FP+FN}$$

(7) $$Precision=\frac{TP}{TP+FP}$$

(8) $$F1-score=\frac{2TP}{2TP+FP+FN}$$

where TN is the number of true-negative samples, TP is the number of true-positive samples, FN is the number of false-negative samples, and FP is the number of false-positive samples.

### 4.2 Ablation experiment

Ablation experiments were used to demonstrate the effects of the DCWP and LR modules, and the results are presented in **Table 5**. The model identification accuracy without the DCWP and LR modules was 68.6%. The introduction of the DCWP module increased the accuracy of the model to 79%, and the introduction of the LR module increased the model accuracy to 97.5%; the model accuracy and F1-score were also significantly improved. The recognition accuracy of the proposed model with the DCWP and LR modules reached 99.3%, which was much higher than the recognition accuracy of the benchmark model. After both the DCWP and LR modules was added to the proposed model, the number of parameters of the model increased to 1.577 M, ensuring very few required parameters while maintaining the performance of image feature extraction from images of diseased tomato leaves.

**Table 5:** Results of the ablation experiment.

DCWP module	LR module	Accuracy	Precision	F1-score	Param (M)
–	–	0.686	0.698	0.609	0.013
√	–	0.790	0.793	0.753	0.780
–	√	0.975	0.971	0.970	0.831
√	√	0.993	0.988	0.987	1.577

"√ " indicates that there is this module (column name) in this column. For example, the "√" in the second and fourth rows of the "DCWP module" column represents the presence of a DCWP module. The "-" indicates the absence of the module (column name) in this column. For example, the "-" in the first and third rows of the "DCWP module" column represents the absence of a DCWP module.

### 4.3 Comparison results of the proposed model with classical CNN models

The proposed LightMixer model was then compared with other classical CNN models, namely AlexNet (Krizhevsky, 2014), ResNext (Xie et al., 2017), ResNet34 (He et al., 2016), ResNet50 (He et al., 2016), VGG16 (Simonyan and Zisserman, 2014), and VGG19 (Simonyan and Zisserman, 2014), in a comparative experiment on the tomato leaf disease dataset. **Table 6** presents the comparative results for the different models. The proposed LightMixer model achieved 99.3% accuracy compared with other classical network models, which exhibited optimal performance. The weighty models of the suboptimal models are ResNext, ResNet34, and ResNet50. The accuracy of the ResNext, ResNet34, and ResNet50 models was stable at 99%. The accuracies of the AlexNet, VGG16, and VGG19 models were 96.2%, 96.6%, and 95.7%, respectively; they were approximately 3% less accurate and exhibited poorer performance than the LightMixer model. In addition, the LightMixer model proposed in this study has the fewest parameters among the many classical CNN models compared. The experimental results showed that the proposed LightMixer model has better performance on the tomato leaf disease dataset than other classical CNN models.

**Table 6:** Comparison of the classical CNN models with the proposed LightMixer model.

Model	Accuracy	Precision	Recall	F1-score	Param (M)	FLOPs(G)
ResNet34	0.992	0.984	0.986	0.985	21.8	3.7
ResNet50	0.993	0.989	0.987	0.988	25.6	4.1
VGG16	0.966	0.958	0.958	0.958	138.4	15.5
VGG19	0.957	0.953	0.948	0.950	143.7	19.6
AlexNet	0.962	0.934	0.940	0.936	61.1	0.7
ResNeXt	0.994	0.989	0.987	0.988	25.0	4.3
**Proposed LightMixer model**	**0.993**	0.986	0.983	0.984	**1.5**	2.2

**Figure 5:**
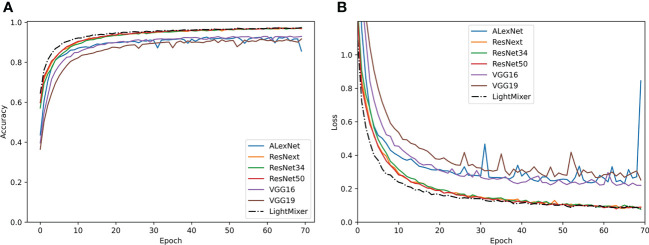
Training curves of the classical CNN models and the proposed LightMixer model. **(A)** Accuracy; **(B)** loss.

**Figure 5** shows the performances of the LightMixer model and other classical CNN models during training, including the accuracy and loss between these models. As shown in **Figure 5A**, the accuracy of the LightMixer model was higher than that of the other models at the initial stage. In addition, the LightMixer model was the first to transition to the smoothing curve and achieved 99% accuracy after the curve is smoothed. In addition to accuracy, the LightMixer model demonstrated strong convergence and generalization during the training process. As shown in **Figure 5B**, the loss curve of the LightMixer model was the first to transition to a smoothed curve and then remained stable. However, this was not the case for most other models, especially the AlexNet model. These comparisons demonstrate the superiority of the LightMixer model.

**Figure 6:**
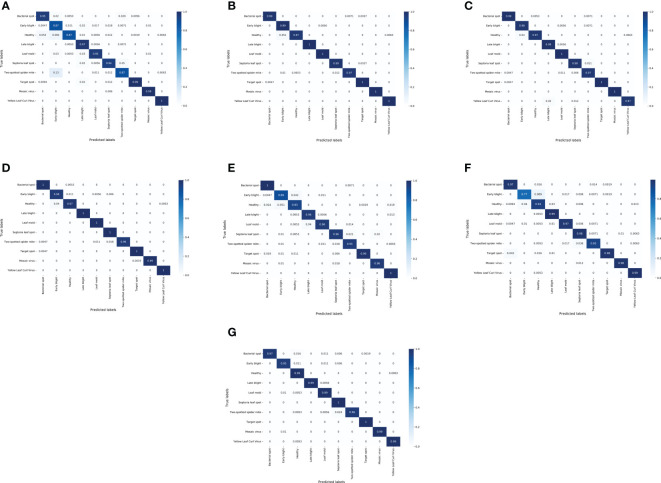
Confusion matrix between the classical CNN models and the proposed LightMixer model. **(A)** ALexNet; **(B)** ResNext; **(C)** ResNet34; **(D)** ResNet50; **(E)** VGG16; **(F)** VGG19; **(G)** LightMixer.

To observe the performance of different models in identifying different classes of tomato leaf diseases, a confusion matrix was used to visualize the classification results on the test set. **Figure 6** shows the confusion matrix of the LightMixer model with other classical CNN models. The vertical axis represents the True Label, and the horizontal axis represents the Predicted Label. The values on the diagonal line represent the ratio of the number of correctly predicted disease samples for that class to the total number of samples for that class of images, and the values on the non-diagonal line represent the fraction of incorrect predictions. The values on the diagonal of the confusion matrix in **Figure 6** **Figure 5G** are all very high, with more than half of the tomato leaf disease classes identified with an accuracy of 99% or higher. The LightMixer model was more than 95% accurate for the other disease categories. The confusion matrices for the ResNext and ResNet50 models were similar. However, the VGG19 model was the least effective in classifying all types of tomato leaf diseases, especially in identifying early blight, with an accuracy of only 77%. The experimental results showed that the LightMixer model can efficiently and accurately classify various types of tomato leaf diseases.

### 4.4 Comparison of the proposed model with the classical lightweight CNN model

**Table 7** shows the identification results of the proposed model compared with those of the classical lightweight CNN model for the tomato leaf disease validation set. LightMixer demonstrated greater accuracy than the compared lightweight CNNs, including DenseNet121 (Huang et al., 2017), EfficientNet (Tan and Le, 2019), MobileNetV2 (Sandler et al., 2018), MobileNetV3 (Howard et al., 2019), ShuffleNetV2 (Ma et al., 2018), SqueezeNet (Iandola et al., 2016) and MNASNet (Tan et al., 2019)Although the Dense-Net121 and LightMixer models have similar accuracies, the LightMixer model requires approximately six times fewer parameters than the Dense-Net121 model. In addition, the proposed LightMixer model has only 1.5 M parameters, which is several to ten times less than the parameters of other lightweight models such as MobileNet V1, V2, and V3. Although the number of parameters of the SqueezeNet model was similar to that of the LightMixer model, the LightMixer model was more accurate. These experimental results demonstrate the advanced performance of the LightMixer model as a lightweight model, which is more suitable for lightweight execution of tomato leaf disease identification tasks.

**Table 7:** Comparison of the classical lightweight CNN models with the proposed LightMixer model.

Model	Accuracy	Precision	Recall	F1-score	Param (M)
DenseNet121	0.993	0.989	0.987	0.988	8
EfficientNet	0.985	0.985	0.982	0.983	7.8
MobileNetV2	0.970	0.963	0.965	0.964	3.5
MobileNetV3	0.985	0.984	0.983	0.984	2.5
ShuffleNetV2	0.988	0.986	0.983	0.985	2.3
SqueezeNet	0.980	0.977	0.975	0.976	1.2
MNASNet	0.959	0.947	0.945	0.945	2.2
**Proposed LightMixer model**	**0.993**	0.986	0.983	0.984	**1.5**

**Figure 7:**
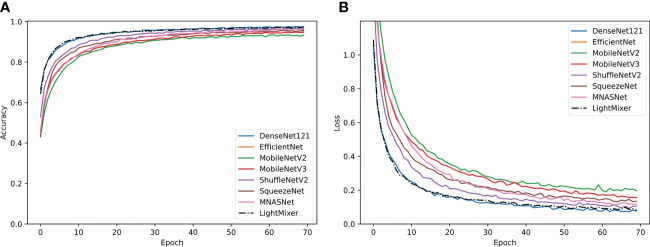
Training curves of the classical lightweight CNN models and the proposed LightMixer model. **(A)** Accuracy; **(B)** loss.

**Figure 7** shows the training curves of the LightMixer model compared with those of other classical lightweight CNN models. Compared with other classical lightweight convolutional networks, the LightMixer model exhibits excellent learning ability during training. As shown in **Figure 7A**, the LightMixer model is one of the first models to transition to a smooth curve. In addition, it has the best accuracy and loss, as well as the least curve fluctuation among the different lightweight CNNs. **Figure 7B** shows the loss curves of different lightweight convolutional networks.

**Figure 8:**
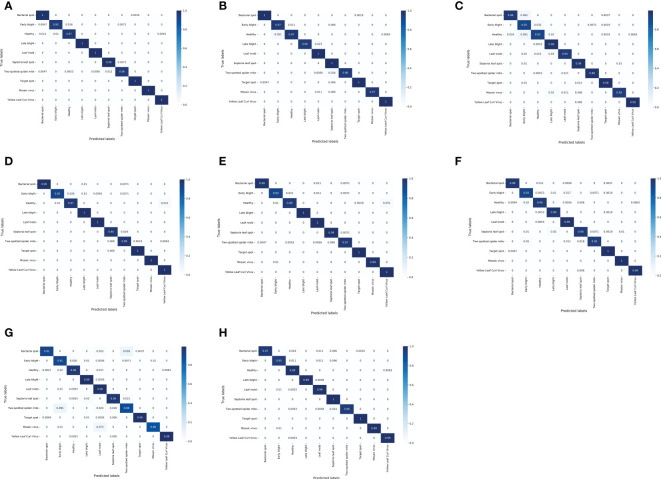
Confusion matrix between the classical lightweight CNN models and the proposed LightMixer model. **(A)** DenseNet121;**(B)** EfficientNet;**(C)** MobileNetV2;**(D)** MobileNetV3;**(E)** ShuffleNetV2;**(F)** SqueezeNet;**(G)** MNASNet; **(H)** LightMixer.

**Figure 8** **Figure 8** shows the confusion matrix of several lightweight CNNs on the test dataset. For the test set of tomato leaf diseases, the MNASNet model showed the worst classification results, where the accuracy of mosaic virus recognition was only 86%. In addition, the SqueezeNet model, which has a similar number of parameters to the LightMixer model, did not show an advantage in terms of classification effectiveness. The SqueezeNet model showed uneven classification accuracy for various types of tomato leaf diseases, with the lowest recognition accuracy of 93% for early blight, whereas the LightMixer model was 95% accurate for this type of disease. These experimental results demonstrate that the LightMixer model maintains its classification advantage for different types of tomato leaf diseases compared with other lightweight models.

## 5 Conclusions

The identification and classification of various tomato leaf diseases by CNN models are highly accurate; however, mainstream CNN models require higher computational and storage costs. In this study, an extremely lightweight CNN model called LightMixer is proposed for the automatic identification of tomato leaf diseases. In the proposed model, LR residual blocks are introduced as one of the main core units for building the backbone network. The architecture of LR residual blocks is designed to achieve better performance while requiring less computation and storage costs. The proposed LightMixer model also has a DCWP module that enhances the feature extraction capability of the network, highlights the nonlinear representation of the model, and compresses the parametric number of the model to enhance its classification capability. The combination of the LR residual block and DCWP module facilitates the ability of the model to utilize the feature map to better capture the disease features and help optimize the performance of the model. Experimental results demonstrate that the proposed model has 99.3% recognition accuracy in diagnosing tomato leaf diseases and has only 1.5 M parameters, which is better than other tested classical models. In the next step, the deployment of the proposed model in mobile or embedded device environments will be our main goal to help farmers accurately identify and detect tomato leaf diseases. Further exploration of the generalizability of the proposed model to detect and identify a variety of other plant diseases will be part of our future plans.

## Data availability statement

Publicly available datasets were analyzed in this study. This data can be found here: https://www.kaggle.com/datasets/abdallahalidev/plantvillage-dataset.

## Author contributions

MT contributed to conception and design of the study. YZ designed and performed the experiment, managed the algorithms and result analysis and wrote the manuscript. ZT participated in the experiments and manuscript revision. All authors contributed to the article and approved the submitted version.

## Supplementary material

Supplementary Figure 1 Samples of tomato diseased leaves. **(A)** Bacterial spot; **(B)** Early blight; **(C)** Healthy; **(D)** Late blight; **(E)** Leaf mold; **(F)** Septoria leaf spot; **(G)** Two-spotted spider mite; **(H)** Target spot; **(I)** Mosaic virus; **(J)** Yellow Leaf Curl Virus.

Supplementary Figure 2 The result after data enhancement. **(A)** Original image; **(B)** Random Image Crop; **(C)** horizontal random flip; **(D)** vertical random flip; **(E)** random rotation.
