# Supplementary figures and images for: LightMixer: A novel lightweight convolutional neural network for tomato disease detection
Source: Front Plant Sci. 2023 May 9;14:1166296. doi: 10.3389/fpls.2023.1166296 (PMC10203629; doi:10.3389/fpls.2023.1166296)

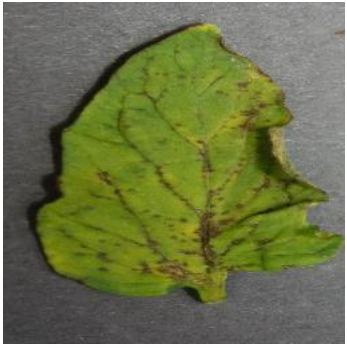

(a)

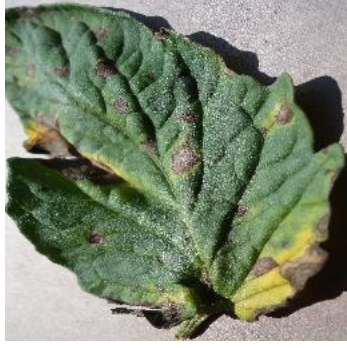

(b)

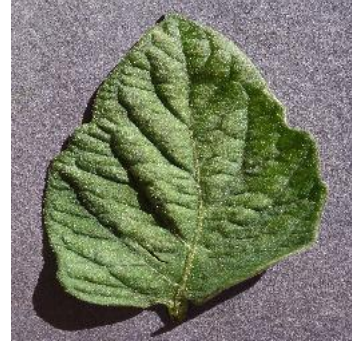

(c)

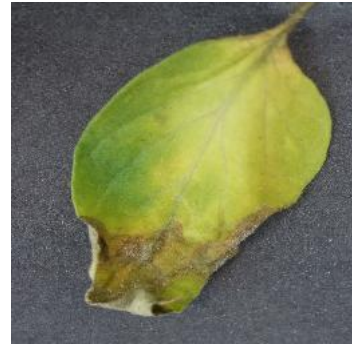

(d)

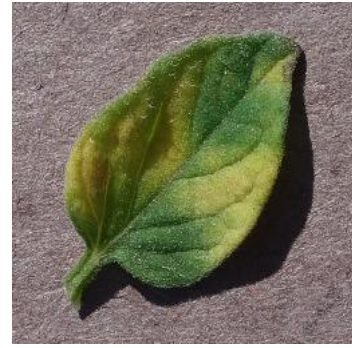

(e)

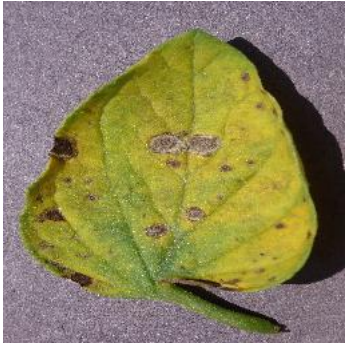

(f)

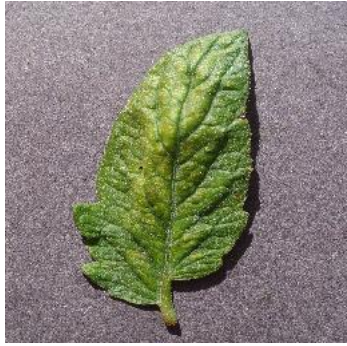

(g)

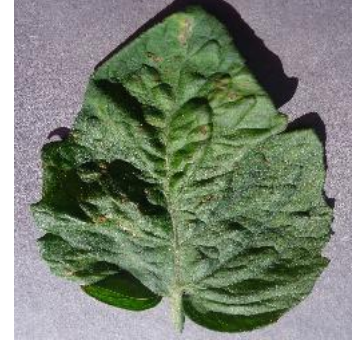

(h)

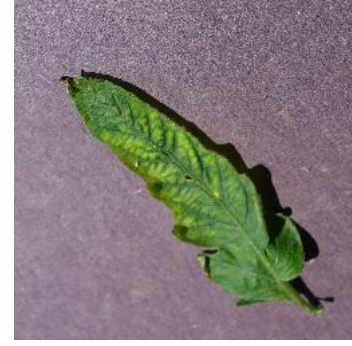

(i)

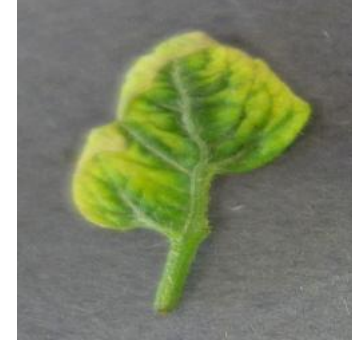

(j)

Supplement: Supplementary Figure 1 — Samples of tomato diseased leaves. (A) Bacterial spot; (B) Early blight; (C) Healthy; (D) Late blight; (E) Leaf mold; (F) Septoria leaf spot; (G) Two-spotted spider mite; (H) Target spot; (I) Mosaic virus; (J) Yellow Leaf Curl Virus. [file Image_1.pdf]

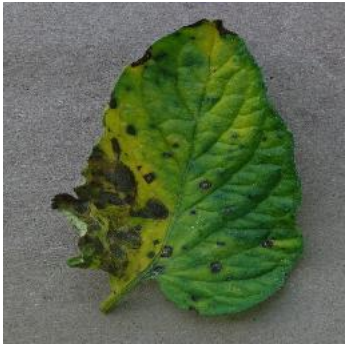

(A)

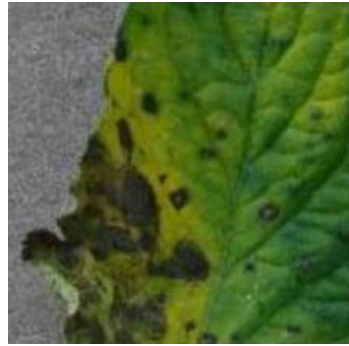

(B)

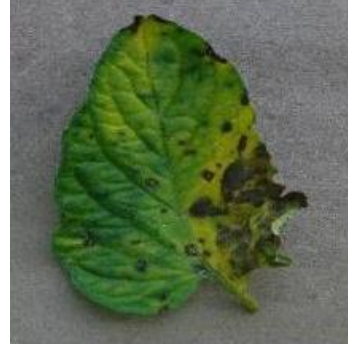

(C)

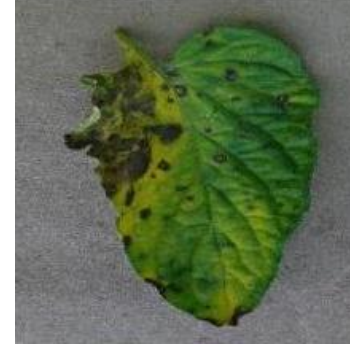

(D)

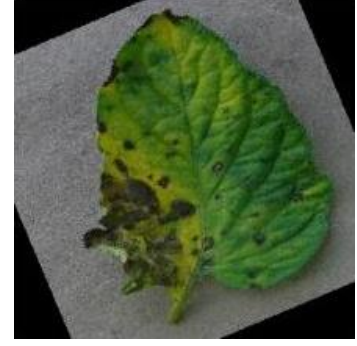

(E)

Supplement: Supplementary Figure 2 — The result after data enhancement. (A) Original image; (B) Random Image Crop; (C) horizontal random flip; (D) vertical random flip; (E) random rotation. [file Image_2.pdf]
